# A Teeth Conference

**Published:** 1920-04-24

**Authors:** 


### A Teeth Conference.

A conference is to be held in Manchester for three days, beginning May 13, on the subject of the prevention of the diseases of the teeth. It is being organised by the Food Education Society, which has been responsible for three previous conferences—one a conference of matrons on the feeding of nurses (1910), one on diet and hygiene, and the third on diet, cookery, and hygiene. In extending its campaign to consideration of teeth diseases it is tackling a most important and difficult problem, and a problem which remains difficult and urgent, as Sir George Newman has said, even after ten years of school medical service.

Education authorities and other interested bodies have been invited to send delegates, and a large attendance of local authorities, medical and dental practitioners, nurses, educationists, heads and housekeepers of schools and other institutions, and of social workers is confidently anticipated. As the subject will be treated in a popular rather than a technical manner, the conference should also have much interest for the general public, and this will come opportunely upon the heels of National Health Week. The Treasurer is Sybil Viscountess Rhondda.

